# A Cooperative Environment for Ventricular Assist Device Development and Application: The Japanese Experience

**DOI:** 10.21470/1678-9741-2022-0349

**Published:** 2022

**Authors:** Carlos Karigyo, Jeison Fonseca, Aron Andrade, Minoru Ono

**Affiliations:** 1 Engineering Center for Circulatory Assistance, Dante Pazzanese Institute of Cardiology, São Paulo, Brazil; 2 Postgraduate Program in Medicine/Technology and Intervention in Cardiology, University of São Paulo, São Paulo, Brazil. E-mail: karigyo@usp.br; 3 Department of Cardiac Surgery, The University of Tokyo, Bunkyo-ku, Tokyo, Japan; 4 Department of Cooperative Unit of Medicine and Engineering Research, The University of Tokyo, Bunkyo-ku, Tokyo, Japan

The first heart transplantation (HTx) in Japan occurred in 1968, about eight months after the world’s first case in South Africa. However, the event fell into controversy regarding brain death and medical indication, leading to the interruption of organ transplantation from brain-dead donors for the next three decades^[1]^. The Japanese Organ Transplant Act came into effect in 1997, and the restart of HTx took place in Osaka, in 1999, under new legislation. Two of the three transplanted patients were bridged by ventricular assist devices (VADs), with the first by a Novacor implantable VAD and the second by a Toyobo paracorporeal VAD^[1,2]^. At that time, Novacor had already finished the official trial (started in 1996), and the first patient was supported for compassionate use by courtesy of Medtronic. The Toyobo VAD, implanted in the second HTx patient, was first applied to a postcardiotomy patient in 1982, and as bridge to transplantation in 1992 by an overseas transfer of a patient from Osaka (Japan) to Houston (United States of America)^[1-4]^. With the revision of the transplant law in 2010, accepting the donation without donor’s written will, the number of HTx rose sharply from 10 per year (before revision) to 44 in 2015 and to 70 in 2019. The cumulative number of HTx in Japan has reached a total of 476 in 2019, with an impressive number of 449 patients bridged by VADs^[1,2]^. The development of VADs started very early in Japan, considering the historical absence of organ donation for HTx until the regulatory law of 1997. At the beginning, research efforts were conducted for the development of extracorporeal VADs, headed by two groups, one at the University of Tokyo (UT) and the other at the National Cardiovascular Center (NCVC)^[1,3]^. The UT project started in the early 1970s and released a pneumatic sack-type pulsatile extracorporeal VAD, in collaboration with Tohoku University, Nippon Zeon, and Aishin Seiki corporations. The pump was first implanted in a postcardiotomy patient in 1980, being the first one to be supported by a VAD in Japan. The Zeon VAD was approved for insurance reimbursement in 1994, and a total of 160 pumps were implanted until its discontinuation in 1998^[1,3]^. In the early 1980s, the NCVC project released a diaphragmatic air-driven extracorporeal pump, being supported by Toyobo Co. (Osaka, Japan). The first clinical implant was performed in a postcardiotomy patient in 1982. As the Zeon pump, the Toyobo VAD was approved for insurance reimbursement in 1994. Since 2012, Nipro Co. (Osaka, Japan) has been managing the device, which changed its name to Nipro VAD^[1,3]^. Both Zeon and Toyobo pumps were originally conceived for profound heart failure patients, primarily for postcardiotomy shock, and were responsible for the beginning of the clinical mechanical circulatory support (MCS) in Japan. Before the restart of the HTx era, a clinical report that covered both devices demonstrated 50% of weaning and 26% of survival rates, showing the promising value of VADs in the acute heart failure therapy^[1,3]^. With the early positive outcomes through extracorporeal VADs and with the imminence of HTx restart, durable and implantable devices were needed to be taken into consideration for a long-term support as a bridge strategy for potential candidates^[1,4]^. The HeartMate-I series (Thoratec, United States of America) were the first implantable pulsatile VADs to be introduced in 1994, with very convincing results in durability and lower complication rates. A clinical trial from 2001 to 2003 was carried out with the HeartMate VE, the second version of the series after the HeartMate IP, with the government approval coming in 2009 for a third version (HeartMate XVE)^[1,4]^. The Novacor (World Heart, Canada) was introduced in 1996 with a clinical trial evaluating it as a long-term device with the support of Medtronic Japan (Tokyo, Japan), and the insurance reimbursement was achieved in 2004. However, the company discontinued its manufacture two years later^[1,4]^. As previously described, the first HTx patient under legislation was bridged by a Novacor VAD^[1]^. During the 1990s, two original VADs were developed in Japan. Under the support of Tokyo Women’s Medical University (TWMU), Waseda, and Pittsburgh Universities, the group conceived a new implantable centrifugal pump named EVAHEART (Sun Medical Technology Research, Japan)^[1,5]^. The centrifugal pump consists in an open vane impeller levitated with an ultrathin layer of circulating water (hydrodynamic levitation)^[5]^. Government approval as a medical device came in 2004, the first human implant was performed at TWMU in 2005, and insurance reimbursement was achieved in 2010^[1,4,5]^. Starting in 2014, a clinical trial from the United States of America reached 191 implants, of which 74 were transplanted. A new small-sized model has been recently released (EVAHEART-2)^[1]^. Another project was originated from the collaboration of TWMU, NTN Co. (Osaka, Japan), and Terumo Corp. (Japan), which developed the DuraHeart in 1994. The centrifugal pump contained a spinning impeller magnetically levitated within the blood path^[1,6]^. In 2004, DuraHeart started European trial and Conformité Européenne mark was obtained in 2007. In Japan, an additional clinical trial started in 2008 and obtained government approval in 2010. In 2017, the device was discontinued for commercial supply^[1]^. HTx and the development of VADs started very early, around the 1960s, in Japan. However, the transplant path was suddenly interrupted for about three decades. During this time, the country developed the first artificial devices for primally postoperative cardiac failure as a temporary measure for self-recovering the heart^[4]^. Posteriorly, multi-institutional efforts from universities and companies worked together to introduce implantable VADs considering the coming era of HTx restart, initially with pulsatile devices, and after, moving to continuous flow VADs. Legislative acts and governmental support were crucial elements for the reestablishment of HTx and progress of MCS therapy in Japan. Furthermore, the acquired experience with different types of VADs provided a better preparedness for the management of these very critical patients already mechanically supported. Currently, the national cooperative environment has progressed to a more autonomous and integrated system, conferring more options for bridge therapies and also destination therapy for those patients who are ineligible for HTx.
